# Fiber-based photoacoustic remote sensing microscopy and spectral-domain optical coherence tomography with a dual-function 1050-nm interrogation source

**DOI:** 10.1117/1.JBO.26.6.066502

**Published:** 2021-06-23

**Authors:** Matthew T. Martell, Nathaniel J. M. Haven, Roger J. Zemp

**Affiliations:** University of Alberta, Department of Electrical and Computer Engineering, Edmonton, Alberta, Canada

**Keywords:** photoacoustic, microscopy, remote sensing, optical coherence tomography, dual-modality, fiber-based

## Abstract

**Significance:** Spectral-domain optical coherence tomography (SD-OCT) offers depth-resolved imaging of optical scattering contrast but is limited in sensitivity to optical absorption. Dual-modality imaging combined with the noncontact absorption contrast of photoacoustic remote sensing (PARS) microscopy can augment SD-OCT applications with specific molecular and functional contrasts in an all-optical, fiber-based platform.

**Aim:** To develop a fiber-based multimodal PARS and SD-OCT imaging system, which efficiently uses a common 1050-nm light source for SD-OCT and PARS interrogation.

**Approach:** PARS microscopy has predominantly utilized a 1310-nm interrogation light source to date. Hence, a recent dual-modality PARS and 1050-nm SD-OCT imaging system required three distinct wavelengths including a 532-nm PARS excitation, necessitating a free-space optical architecture with discrete subsystems. Here, we validate the first use of a 1050-nm interrogation wavelength for PARS. This enables the transition to fiber-based interferometry as is standard in modern SD-OCT systems, though infeasible with inclusion of an additional 1310-nm wavelength. PARS interrogation functionality is integrated using a broadband optical circulator.

**Results:** Dual-modality imaging is demonstrated in carbon fiber phantoms and a mouse ear *in vivo*. SD-OCT provided a 4.5-μm lateral resolution, 8.8-μm axial resolution in air, and >101  dB of sensitivity, and PARS contributed 532-nm optical absorption contrast with a 47-dB SNR, and lateral and axial resolutions of 2.4 and 35  μm, respectively. Total interrogation power was reduced from 90% to 58% of the ANSI limit compared to a previous three-wavelength approach.

**Conclusions:** Adapting PARS to use the 1050-nm SD-OCT light source for interrogation enabled implementation of a fiber-based dual-modality system configuration, with image quality maintained. This will facilitate development of potential applications demanding handheld, catheter-based, or endoscopic form factors.

## 1 Introduction

Optical coherence tomography (OCT) is a modality that generates depth-resolved images of the optical scattering intensity within a sample.^1^ Functioning on the basis of low-coherence interferometry, it is capable of providing μm-order axial resolution, determined inversely by the spectral bandwidth of the light source. The utility of OCT has been proven in several clinical and preclinical applications, particularly in ophthalmology. Although OCT offers remarkable sensitivity to backscattered light, its ability to reveal optical absorption is relatively limited, and scattering contrast alone provides poor specificity for differentiating salient features in many tissues. While polarization-sensitive,^2^ flow-sensitive,^3^ and spectroscopic^4^ methods have been developed to add functional information to structural images, OCT in general is not an ideal platform for imaging endogenous molecules or exogenous agents. Hence, several multimodal imaging systems have been reported, combining OCT with the complementary contrast of other imaging mechanisms including fluorescence,^5^ photoacoustic, and nonlinear microscopies.^6^^–^^8^

Photoacoustic microscopy (PAM) methods form a particularly effective combination with OCT, augmenting scattering with optical absorption contrast.^9^^–^^12^ Conventionally, optical resolution PAM (OR-PAM) uses an ultrasound transducer to detect acoustic waves generated by absorption of a pulsed excitation laser scanned over the sample. This technique is versatile, as the excitation wavelength can be targeted to the absorption spectra of various chromophores of interest, to visualize application-specific features. In a recently developed, all-optical approach called photoacoustic remote sensing (PARS) microscopy, the acoustic detection is replaced with an interrogation light source cofocused with the excitation beam.^13^ Absorption-induced modulation of the sample refractive index is then detected using reflectometry at the interrogation wavelength. This eliminates the need for mechanical coupling to the sample, though the acoustic depth ranging of PAM is relinquished. Alternatively, PARS offers high-resolution, noncontact optical absorption imaging in reflection-mode, with depth discrimination instead provided by optical sectioning.^14^

In recent work, we reported an all-optical, dual-modality imaging system that integrated PARS microscopy with spectral-domain optical coherence tomography (SD-OCT).^15^ This proved effective for simultaneous, coregistered imaging of complementary optical scattering and label-free absorption contrasts, with noncontact operation at cm-scale working distances. However, this first-generation system has technical limitations that may inhibit future translation.

Previous reports on PARS imaging predominantly used a 1310-nm interrogation wavelength,^13^^,^^14^^,^^16^ with a single exception involving 1550-nm interrogation.^17^ Therefore, we elected to adopt this 1310-nm interrogation wavelength in our initial multimodal system given that 1050-nm PARS interrogation had not been investigated. Hence, the system required the use of three distinct light sources: the 1310-nm PARS interrogation source, a 1050-nm low-coherence OCT source, and a 532-nm pulsed excitation laser. Aside from excess optical power delivered to the sample and increased system costs, the inclusion of additional wavelengths constrains the optical components that may be utilized in the system. This can restrict the flexibility of the approach for molecular imaging if the optimal excitation wavelength cannot be accommodated. The use of three wavelengths also requires the complexity of optically coaligning three beams. The PARS excitation and interrogation beams must be precisely cofocused to generate the PARS signal, whereas coregistration of the dual modalities relies on additionally aligning the OCT beam. Moreover, integrating several wavelengths into the beam requires additional dichroic optics, which can introduce spurious reflector artifacts into SD-OCT images.

In modern SD-OCT systems, the use of a fiber-based interferometer has become the standard owing to reduced need for alignment, robustness to environmental vibrations, maintenance-free operation, and compact form factors. The use of this configuration in the previous multimodal system iteration was infeasible, however, due to the inclusion of an additional 1310-nm PARS interrogation source. A free-space optical architecture was necessitated for compatibility with the multiple different wavelengths used. This free-space setup is impaired by its bulk, limited portability, and excessive alignment demands compared to a fiber-based setup. A modular design that separates an independent, fiber-tetherable scanning head from the light sources, detectors, and other core hardware can be advantageous. Such a system offers simpler interfacing with existing imaging devices to facilitate translation to clinical settings and is adaptable to applications requiring handheld, endoscopic, or catheter-based form factors.

In this work, we develop an improved dual-modality PARS microscopy and spectral-domain OCT system configuration to address the limitations of the previous approach. In this case, a single 1050-nm superluminescent diode (SLD) functions as both the low-coherence OCT light source and the PARS interrogation source, which represents the first report investigating PARS interrogation at 1050 nm. From this exploratory work, experimental evidence validating use of this interrogation wavelength option will offer additional flexibility in the system design of future PARS microscopy platforms, as it may enable improved optimization of excitation and interrogation wavelength combinations subject to the practical constraints of optical components. An additional motivating factor for our proposed architecture is to minimize chromatic aberrations when cofocusing excitation and interrogation light through the objective lens. Since 1050 nm is closer to the 532-nm excitation wavelength than a 1310-nm source, we expect that longitudinal chromatic aberration disparity will be reduced. The dual-functionality utilization of the existing 1050-nm SLD enables elimination of the additional 1310-nm light source, reducing system complexity and facilitating a transition to the fiber-optic implementation of the OCT interferometer that is considered the current standard. A fiber-based PARS interrogation pathway is then integrated into the interferometer using an optical circulator. This optical architecture is interfaced to a fiber-tetherable scanning head, which can be adapted to an application-specific scanning device when translated to clinical settings. Our previous PARS-OCT system could not achieve a fiber-based implementation because single-mode, high numerical aperture (NA) fiber couplers and circulators for the 1310-nm wavelength are not typically single-mode for a 1050-nm wavelength. In addition, a distinction presented here in comparison to previous work is the in-line combination of interferometric OCT with noninterferometric PARS by strategic placement of the reference mirror optical path length, avoiding interference between the modalities. Complementary dual-contrast imaging with the enhanced system is demonstrated for comparing images to the previous generation dual-modality system.

## 2 Materials and Methods

### 2.1 Optical Setup

**Fig. 1:**
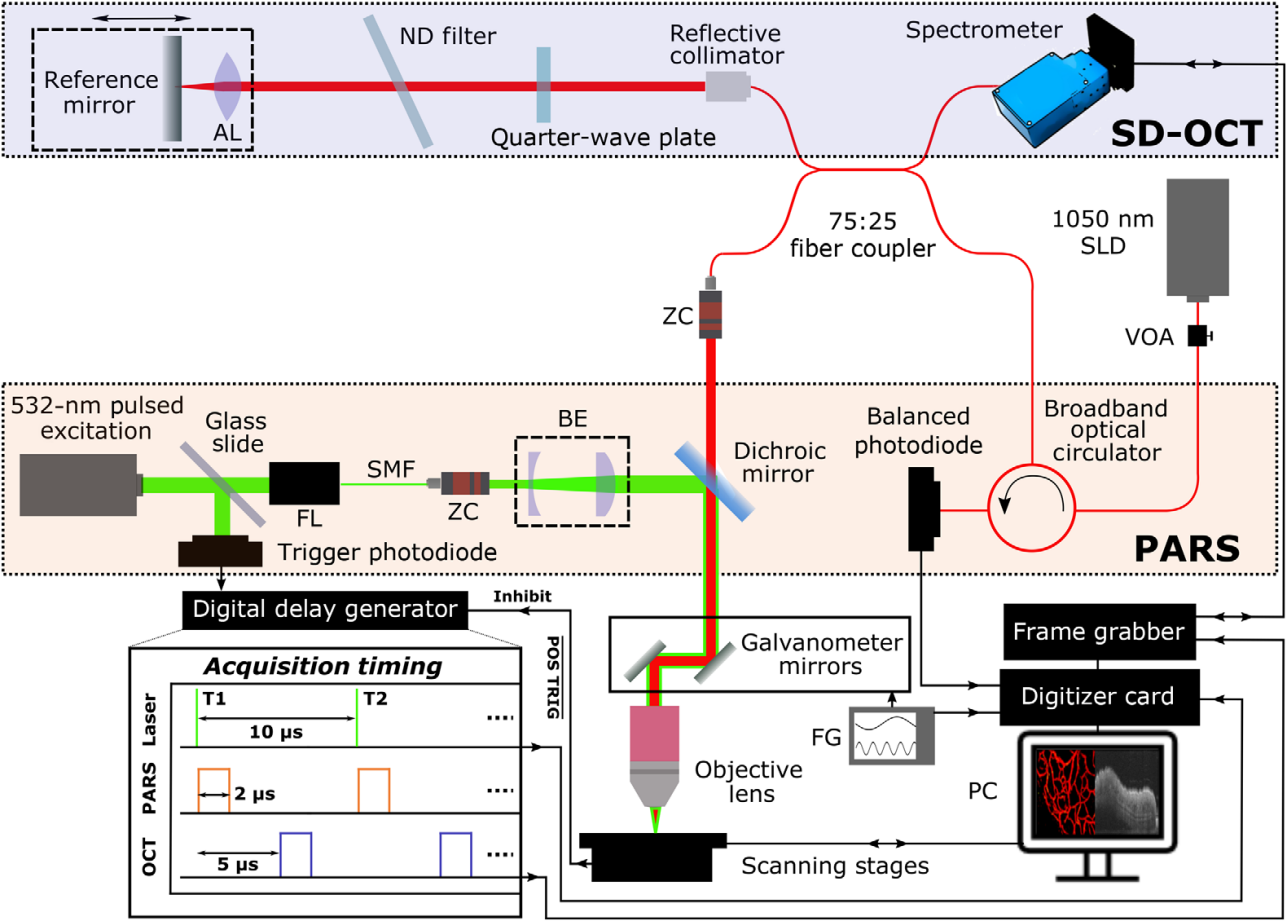
Dual-modality optical system. AL, aspheric lens; BE, beam expander; FG, function generator; FL, fiber launch; ND, neutral density; SMF, single-mode fiber; VOA, variable optical attenuator; ZC, zoom collimator.

A diagram of the fiber-based dual-modality system is shown in Fig. 1. The dual-purpose SLD light source (SLD1050S-A60, Thorlabs) emits at a center wavelength of 1045 nm, with a maximum 67.7 mW of amplified spontaneous emission power and an optical bandwidth of 77.4 nm when driven at 1 A. Operating the SLD device at this constant current is necessary to maintain the full emission bandwidth for optimal axial resolution. However, as the operating current also determines the emitted optical power, a variable attenuator (VOA1064-APC, Thorlabs) was used to reduce the incident power on the sample to 4 mW.

The free-space PARS interrogation path in previous work relied on the polarized property of the light source and used a quarterwave plate in conjunction with a polarizing beam splitter to redirect light reflected from the sample to the detection photodiode. To achieve this functionality in a fiber-based system without restricting the optical bandwidth, and consequently the OCT axial resolution, we used a broadband optical circulator (ABPICIR-1050-H6-L-10-FA, OF-Link Communications) with a wavelength specification of 1050±50  nm. This component delivers light from the SLD source toward the sample path, collects the PARS interrogation light returning from the sample, and directs it to a 75-MHz balanced photodiode (PDB425CAC, Thorlabs). The balanced photodiode was employed due to its AC-coupled RF output and integrated high transimpedance gain amplifier, with only one optical input utilized. The circulator additionally serves as an optical isolator to protect the SLD device from damaging backreflections.

The 1050-nm PARS interrogation light forwarded from the sample path port of the circulator was integrated into a fiber-based Michelson interferometer for SD-OCT, implemented using a 2×2 wideband fiber optic coupler (TW1064R3A2B, Thorlabs). In the forward direction, the coupler splits 75% of the optical power received from the circulator toward the sample and directs the remaining 25% to the SD-OCT reference path. This reference path consisted of a reference mirror mounted on a single-axis translation stage for path length variation to set the zero delay point, a neutral density filter to avoid detection saturation by the strong mirror reflection, and an achromatic quarterwave plate allowing some coarse adjustment of polarization. For the return path of the coupler, 75% of the light from the sample were directed to the PARS interrogation photodiode via the circulator and 25% to the SD-OCT spectrometer. As the returning interrogation light must be shared between the dual-modalities in this system configuration, the coupling ratio was selected to bias the reflected optical power in favor of PARS detection, which is considerably less sensitive than SD-OCT. The SD-OCT spectrometer (Cobra 1050, Wasatch Photonics) contains a 2048-pixel InGaAs array linescan camera, offering a 110-nm wavelength detection bandwidth, >63  dB dynamic range in the digitized signal intensity, and a 50-pm spectral resolution.

In the sample path of the interferometer, light was coupled into free-space using zoom collimators (ZC618FC-A and ZC618APC-B, Thorlabs) to allow adjustment of the beam diameter and thereby the effective NA of the optical focusing. A 532-nm ns-pulsed fiber laser (GLP-10, IPG Photonics) provided 60-nJ photoacoustic excitation pulses and was integrated into the near-infrared beam path using a dichroic mirror (DMLP900R, Thorlabs). A partial reflection of the excitation light onto a 350-MHz Si photodiode (DET10A, Thorlabs) served as a trigger for data acquisition. The up to 100-kHz excitation laser repetition rate used in this work was 5× that previously reported,^15^ allowing rapid simultaneous data acquisition given up to 147-kHz line rate capability of the spectrometer camera. The combined beam was focused onto the sample with a 10×, 0.26 NA apochromatic objective lens (M Plan Apo NIR 10x, Mitutoyo).

In spectral-domain OCT, the fixed position of the reference mirror must be set such that the zero delay point is outside the sample being imaged, to avoid the mirror image artifact known to arise from the Hermitian symmetric Fourier transform of the real-valued interferometric signal acquired by the spectrometer.^18^ Thus, while the balanced photodiode in our integrated fiber-based system receives light from both the sample and OCT reference mirror, the path lengths of the reference mirror and any sample reflector differ by several coherence lengths, and therefore will not interfere coherently in the time-domain photodiode signal with a stationary reference mirror.

### 2.2 Sample Scanning and Data Acquisition

Fast, small field-of-view (FOV) optical sample scanning was provided by a galvanometer mirror system (GVS012, Thorlabs), with the excitation laser operating at 100 kHz. Widefield mechanical scanning of samples was performed using stepper motor stages for the x- and y-axes (Micos PLS-85 with C-663 Controller, Physik Instrumente). A constant velocity sampling strategy was employed for the fast axis. While this stage moved at a fixed speed of 20  mm/s through the scan range, active low triggers were output from the controller at regular intervals of 2  μm of travel. As the controller drives the stage with a trapezoidal velocity profile, a 2-mm buffer of motion without data acquisition was added to each side of the scan range to accommodate the acceleration of the stage, avoiding triggering when the lateral spacing is irregular. Each complete fast axis B-scan was followed by a 2-μm step of the slow axis, generating a raster scan pattern over the imaged region. A stepper motor stage for the z-axis (X-VSR20A-E01, Zaber Technologies) was used for precise focus adjustment and stability but remained stationary during scans. For stage scanning, the 20-mm/s velocity limit required an excitation pulse repetition rate of 20 kHz, though the 100-kHz A-line rate used with galvanometer mirror scans could be matched if faster stages were employed.

The interlaced simultaneous data acquisition scheme was coordinated using a digital delay generator (DG645, Stanford Research Systems), as outlined in the timing diagram of Fig. 1. The 532-nm pulsed laser pick-off measured by the trigger photodiode was input to the digital delay generator (DDG) to act as a master trigger signal. On detecting an excitation pulse, the DDG outputs a signal to trigger a PCIe digitizer card (CSE1242, GaGe) to capture a 320-ns window of the PARS interrogation photodiode signal immediately following excitation, when the reflectance perturbation occurs. This consists of 64 samples at 200  MS/s of the balanced photodiode RF output, passively analog filtered from 1.8 MHz (EF509, Thorlabs) to 11 MHz (BLP-10.7+, Mini-Circuits Inc.) to extract the PARS modulation. Following a 5-μs delay to avoid the excitation effect, the DDG outputs a trigger signal to a frame grabber (PCIe-1433, National Instruments) controlling the spectrometer camera, to capture the spectral data for a single SD-OCT A-scan. An exposure time of 6.9  μs was used for each SD-OCT A-line. The active low logic level trigger output ($\overset{¯}{\mathrm{POS}\;\mathrm{TRIG}}$ in Fig. 1) from the fast axis stage controller was input to the DDG as an inhibit signal. Thus, the excitation pulses only trigger the dual-modality data acquisition sequence when the delay generator is uninhibited: that is, following each 2-μm step size of the stage motion. Scans sampled up to 200,000 lateral positions in total, each corresponding to one pixel in a two-dimensional (2D) PARS *en-face* image and one A-scan in depth within a three-dimensional (3D) SD-OCT tomogram. The interlaced scanning approach additionally serves to intrinsically coregister the dual-modality images in the lateral directions. As a representative example of the achievable scan speed, capturing an FOV of ∼1.6  mm×0.5  mm required a total acquisition time of ∼3.7  min, currently limited by the stepper motor stages. Utilizing faster stages offering similar controller positioning signals to implement this scanning approach could improve the scan time to be limited by the excitation laser pulse repetition rate.

The signal processing used for generating PARS images and SD-OCT images is described in detail in our previous work.^13^^,^^15^ SD-OCT reconstruction included the standard steps of background subtraction, spectral reshaping, λ to k-domain uniform resampling, dispersion compensation, and Fourier transformation. The Delaunay triangulation-based interpolation algorithm was only required for galvanometer mirror scans featuring scattered lateral positions acquired on sinusoidal scan trajectories, whereas stage scanning produces an inherently Cartesian grid-based sampling pattern. Gaussian smoothing was used to reduce speckle noise in OCT images, with negligible loss in detail, as the scan step size resulted in subresolution lateral oversampling by a factor of >2×. A Frangi vesselness filter was applied to *in vivo* PARS images, as in previous work.^14^

## 3 Results

### 3.1 Phantom Imaging Experiments

**Fig. 2:**
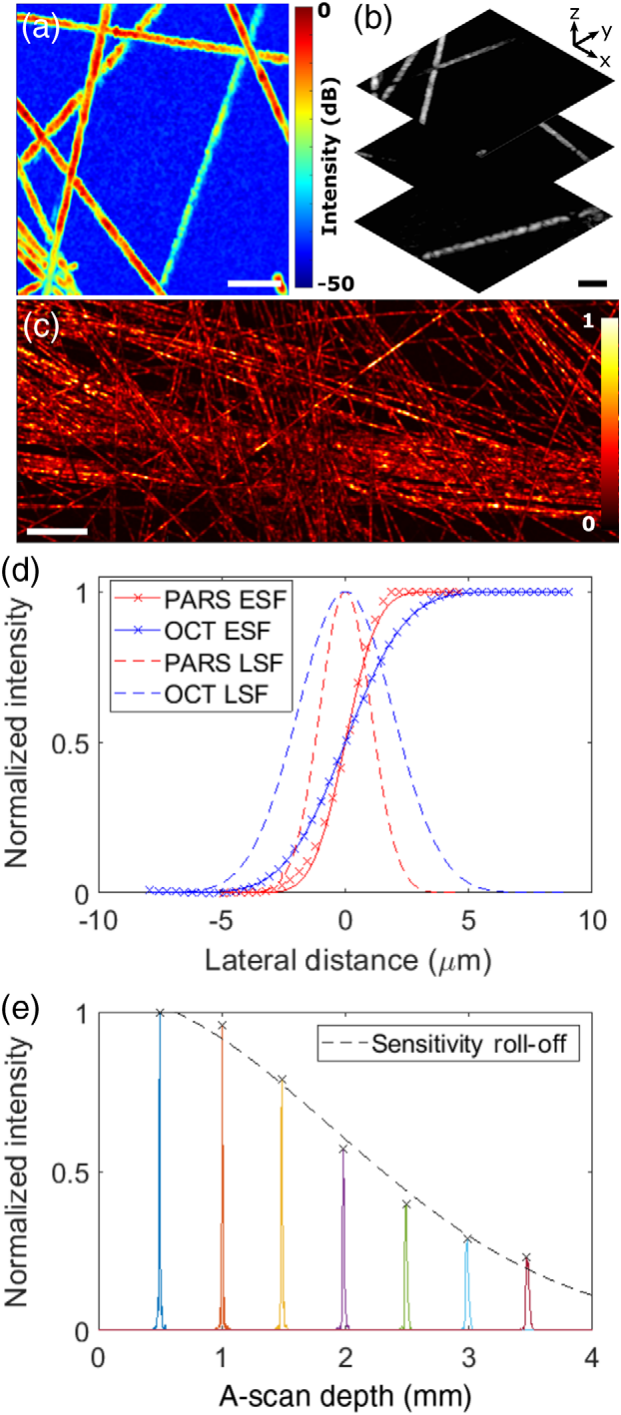
Phantom imaging experiments and system characterization. (a) Optically scanned PARS and (b) SD-OCT images of 7-*μ*m carbon fibers. Scale bars: 50  μm. (c) Stage-scanned PARS image of a dense fiber network. Scale bar: 200  μm. (d) Dual-modality lateral ESF and LSF. (e) SD-OCT axial point spread functions for a mirror at varied depths, indicating axial resolution and roll-off.

For initial validation studies, we imaged networks of 7-*μ*m carbon fibers, which act both as an absorbing target at the 532-nm excitation wavelength, and a scattering target at 1050 nm. Results are shown in Figs. 2(a)–2(c). Concordance is clearly observed between the dual-modality images. PARS absorption contrast imaging offers a 2D projection of the carbon fibers within the optical depth-of-focus. SD-OCT is additionally capable of resolving the projection of carbon fibers observed within the PARS image into their individual depths, given the coherence-gated axial resolution.

### 3.2 System Characterization

Using phantom imaging results, we additionally performed a comprehensive study of the figures of merit for the dual-modality system. To quantify the lateral resolutions, transverse profiles of carbon fibers were used to find edge spread functions (ESFs) for each modality [Fig. 2(d)]. An error function was fit to each ESF data set, with differentiation yielding the corresponding Gaussian line spread functions (LSFs). From this analysis, we determined a PARS full-width at half-maximum (FWHM) lateral resolution of 2.4  μm and an OCT FWHM lateral resolution of 4.5  μm. The PARS lateral resolution is comparable to prior work, whereas the SD-OCT resolution is an improvement over the 6.1  μm previously achieved.^15^ By mechanically scanning a carbon fiber target in depth using a z-axis stage with the lateral position fixed, we estimated an FWHM axial resolution of 35  μm for the PARS microscopy modality, arising from the optical focusing.

The coherence-gated axial resolution of the SD-OCT subsystem was characterized using a mirror as the sample [Fig. 2(d)]. At a depth of 0.5 mm beyond the zero-delay point, we measured an FWHM axial resolution of 8.8  μm in air, corresponding to 6.4  μm in tissue with a refractive index of 1.38. This represents an improvement of ∼1  μm over previous work given the use of a wider emission bandwidth SLD source with a shorter center wavelength.^15^ However, the theoretical axial resolution limit of this light source could not be realized due to a shift between the SLD power spectral density and spectrometer wavelength range. As expected, we observe an axial resolution decay with depth due to breakdown of the λ−k interpolation process required in the image reconstruction, with the FWHM of the axial point spread function measured as >17  μm beyond 2 mm in depth. The sensitivity roll-off with depth, a well-understood limitation of the spectral-domain OCT approach emerging from the spectrometer sampling process,^19^ was observed by measuring the coherence function for the mirror sample at various optical delays [Fig. 2(e)]. This was accomplished by translating the reference mirror with the sample mirror fixed, to avoid signal intensity variation associated with the focused Gaussian beam profile. A low roll-off characteristic of 4.4 dB over 2 mm in depth was determined.

PARS imaging was achieved with a signal-to-noise ratio 47 dB [Fig. 2(a)], defined as the mean signal from a target relative to the background noise standard deviation. Using a mirror as the sample, the sensitivity of the SD-OCT system was determined to be >101  dB. This measurement was performed with 60 dB of absorptive neutral density filter attenuation added to the sample arm as described in Ref. 20, as in general the sensitivity of SD-OCT exceeds the dynamic range of the detection hardware. To optimize the SD-OCT SNR, the reference path power was attenuated such that the receiver noise variance of the spectrometer camera was approximately equal to that of the relative intensity noise originating from the SLD light source.^21^

### 3.3 *In Vivo* Validation

**Fig. 3:**
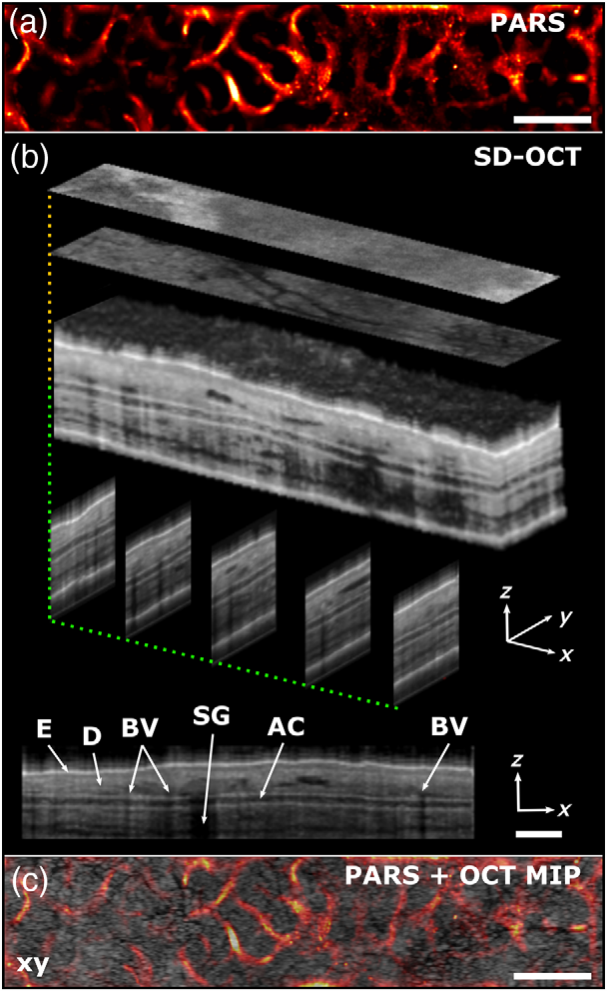
*In vivo* mouse ear imaging. (a) 2D PARS *en-face* image. (b) Volumetric SD-OCT imaging with cross-sectional views through each orthogonal plane. (c) Overlay image of PARS absorption contrast and a maximum intensity projection of the OCT volume data. Scale bars: 200  μm. AC, auricular cartilage; BV, blood vessel; D, dermis; E, epidermis; SG, sebaceous gland.

The *in vivo* performance of the system was evaluated by imaging a hairless mouse ear (SKH1 Elite, Charles River Laboratories), with results shown in Fig. 3. Experimental procedures were carried out in conformity with the laboratory animal protocol approved by the University of Alberta Animal Use and Care Committee. The complementary nature of the contrast mechanisms is evident. PARS generates hemoglobin absorption-based *en-face* imagery of superficial microvasculature, whereas OCT provides a 3D scattering intensity profile revealing dermal structures including the epidermis, dermis, auricular cartilage, and sebaceous glands. While small blood vessels are not readily visible in structural OCT images without applying intensity or phase-based flow detection techniques,^3^ they can produce shadows masking underlying depths (Fig. 3). The 1050-nm SLD light source provides >1 mm penetration depth for SD-OCT, providing a full view through the depth of the mouse ear (Fig. 3). Though the superficial mouse ear vasculature imaged in this study is found at up to ∼300  μm in depth, a previous report has shown that a lateral resolution and depth trade-off can be implemented, with the excitation depth-of-focus extended, and the interrogation beam focus thereby determining lateral resolution.^14^ In this configuration, PARS imaging at up to 1.2 mm *in vivo* has been demonstrated. Currently, it is difficult to assess whether the system is sensitive enough to visualize capillary networks. We observe from our images that vessels have separations of less than ∼100  μm. Given an oxygen diffusion distance of ∼50  μm, this vessel separation suggests that some capillaries may be visualized.

### 3.4 Laser Safety Considerations

The following skin exposure laser safety calculations are based on the analysis described in the supplementary material of Ref. 13. The excitation pulse energy for PARS imaging was set as 60 nJ. Assuming the focal spot is located ∼150  μm below the sample surface, we estimate a surface $1/e^{2}$ spot diameter of 78  μm for the 0.26 NA objective lens. This corresponds to surface laser fluence of $∼1.3\;{\mathrm{mJ}/\mathrm{cm}}^{2}$, which is below the single pulse maximum permissible exposure (MPE) limit of $20\;{\mathrm{mJ}/\mathrm{cm}}^{2}$ established by the American National Standards Institute (ANSI).^22^ Note that the surface fluence in this work exceeds that in previous reports,^13^ due to the lower-NA optical focusing used here to accommodate the SD-OCT modality, which demands an extended depth-of-focus. Given our sample scanning parameters, we additionally estimate that each surface spot size is exposed to a train of 78 spatially overlapping pulses, for a total of ∼3.9  ms on average. ANSI additionally defines a group MPE of $1.1\times C_{A}\times t^{0.25}=275\;{\mathrm{mJ}/\mathrm{cm}}^{2}$ for the full pulse train, where the correction factor $C_{A}$ is unity for a 532-nm laser.^22^ In our case, this corresponds to an average fluence limit of $∼3.5\;{\mathrm{mJ}/\mathrm{cm}}^{2}$ per pulse. Thus, our system operated safely with a surface fluence at ∼37% of this limit. Ideally, the laser would be operated at a 10-kHz repetition rate to match 2  μm lateral sampling at the maximum stage velocity of 20  mm/s, though 20 kHz is the lower limit for the laser used in this work. This hardware limitation exposed the sample to excess excitation pulses. The system could therefore in principle operate at ∼19% of the ANSI average MPE limit.

The continuous-wave OCT/PARS interrogation beam optical power was fixed at 4 mW for the duration of sample scanning. At the 1050-nm wavelength of this light source, a Gaussian beam FWHM focal spot size of ∼4.5  μm located ∼150  μm below the sample surface would produce an estimated surface $1/e^{2}$ spot diameter of ∼38  μm for the objective lens utilized. Using our scanning approach, we estimate an exposure time for each surface spot size of ∼1.9  ms. The ANSI guidelines for a CW source indicate an average skin exposure MPE of $1.1\times C_{A}\times t^{-0.75}=604\;{W/\mathrm{cm}}^{2}$, where $C_{A}=5$ at this wavelength. For the given surface spot size, this yields a 1050-nm optical power MPE of 6.8 mW. Hence, the presented system was operated safely at only ∼58% of this limit.

Our previous system architecture additionally included a co-scanned 1310-nm PARS interrogation beam at an incident optical power of 5 mW.^15^ A similar calculation assuming a 78-μm surface spot diameter (confocal with PARS excitation) indicates an average MPE for the 1310-nm interrogation beam of 16 mW. For this dual-beam setup, ANSI limit compliance requires $\frac{P_{1050\;\mathrm{nm}}}{{\mathrm{MPE}}_{1050\;\mathrm{nm}}}+\frac{P_{1310\;\mathrm{nm}}}{{\mathrm{MPE}}_{1310\;\mathrm{nm}}}\leq 1$.^22^^,^^23^ In practice, our previous system achieved $\frac{4\;\mathrm{mW}}{6.8\;\mathrm{mW}}+\frac{5\;\mathrm{mW}}{16\;\mathrm{mW}}=0.9$. Thus, while that system operated slightly below the ANSI limit, the improved dual-function 1050-nm light source setup uses incident optical power more efficiently. A margin remains to increase the incident optical power up to 6.8 mW within the ANSI limit while simultaneously enhancing the PARS and SD-OCT sensitivity. However, the flexibility of individually adjusting the optical power for each modality is forfeited.

## 4 Discussion and Conclusions

We have developed a significantly improved dual-modality imaging system for integrating the noncontact, label-free absorption contrast of PARS microscopy with complementary and coregistered scattering contrast from spectral-domain OCT. By eliminating the 1310-nm interrogation light source of the prior generation system and adapting the 1050-nm OCT SLD source to additionally function as PARS interrogation, we have reduced the complexity and costs of the system. Moreover, this reduced the total optical power incident on the sample by 5 mW over previous work^15^ while maintaining comparable imaging performance. Using a fiber coupler to implement the OCT interferometer and integrating the PARS interrogation path using a wideband optical circulator, we have implemented a fiber-tetherable system architecture that should simplify future translation to clinical applications, especially those demanding portability or compact form factors. The current scanning module can be replaced with an application-specific imaging probe in these cases. An additional benefit realized is superior alignment stability, an important consideration in a system that still requires the cofocusing of two beams. The presented system additionally uses lateral stage scanning that affords the use of a higher NA objective lens, with improved OCT lateral resolution compared to the 11.4-μm resolution previously achieved using a telecentric scan lens.^15^ This however comes at the cost of reduced imaging speed compared to galvanometer scanning. In future work, inclusion of multiple excitation wavelengths may enable functional imaging including estimation of the oxygen saturation of hemoglobin. When combined with the flow estimation abilities of OCT, this could lead to oxygen metabolic imaging capabilities. The system enhancements realized here will be further explored in future work involving SD-OCT combined with virtual histological imaging using ultraviolet PARS microscopy, OCT-guided dynamic focusing for contour scanning,^12^ and applications to oncology, dermatology, ophthalmology, cardiology, and gastroenterology.

## References

[1] Huang D. et al., “Optical coherence tomography,” Science 254(5035), 1178–1181 (1991). SCIEAS 0036-8075 10.1126/science.1957169 1957169 PMC4638169

[2] De Boer J. F. Milner T. E., “Review of polarization sensitive optical coherence tomography and stokes vector determination,” J. Biomed. Opt. 7(3), 359–372 (2002). JBOPFO 1083-3668 10.1117/1.1483879 12175285

[3] Zhang A. et al., “Methods and algorithms for optical coherence tomography-based angiography: a review and comparison,” J. Biomed. Opt. 20(10), 100901 (2015). JBOPFO 1083-3668 10.1117/1.JBO.20.10.100901 26473588 PMC4881033

[4] Morgner U. et al., “Spectroscopic optical coherence tomography,” Opt. Lett. 25(2), 111–113 (2000). OPLEDP 0146-9592 10.1364/OL.25.000111 18059799

[5] Yuan S. et al., “Co-registered optical coherence tomography and fluorescence molecular imaging for simultaneous morphological and molecular imaging,” Phys. Med. Biol. 55(1), 191 (2009). PHMBA7 0031-9155 10.1088/0031-9155/55/1/011 PMC2951762 20009192

[6] Vinegoni C. et al., “Nonlinear optical contrast enhancement for optical coherence tomography,” Opt. Express 12(2), 331–341 (2004). OPEXFF 1094-4087 10.1364/OPEX.12.000331 19471542

[r7] Marks D. L. Boppart S. A., “Nonlinear interferometric vibrational imaging,” Phys. Rev. Lett. 92(12), 123905 (2004). PRLTAO 0031-9007 10.1103/PhysRevLett.92.123905 15089675

[8] Vinegoni C. et al., “Integrated structural and functional optical imaging combining spectral-domain optical coherence and multiphoton microscopy,” Appl. Phys. Lett. 88(5), 053901 (2006). APPLAB 0003-6951 10.1063/1.2171477

[9] Li L. et al., “Three-dimensional combined photoacoustic and optical coherence microscopy for in vivo microcirculation studies,” Opt. Express 17(19), 16450–16455 (2009). OPEXFF 1094-4087 10.1364/OE.17.016450 19770860 PMC2855548

[r10] Jiao S. et al., “Simultaneous multimodal imaging with integrated photoacoustic microscopy and optical coherence tomography,” Opt. Lett. 34(19), 2961–2963 (2009). OPLEDP 0146-9592 10.1364/OL.34.002961 19794782 PMC2883610

[r11] Song W. et al., “A combined method to quantify the retinal metabolic rate of oxygen using photoacoustic ophthalmoscopy and optical coherence tomography,” Sci. Rep. 4, 6525 (2014). SRCEC3 2045-2322 10.1038/srep06525 25283870 PMC4185377

[12] Dadkhah A. et al., “Integrated multimodal photoacoustic microscopy with OCT-guided dynamic focusing,” Biomed. Opt. Express 10(1), 137–150 (2019). BOEICL 2156-7085 10.1364/BOE.10.000137 30775089 PMC6363202

[13] Hajireza P. et al., “Non-interferometric photoacoustic remote sensing microscopy,” Light Sci. Appl. 6(6), e16278 (2017). 10.1038/lsa.2016.278 30167263 PMC6062239

[14] Reza P. H. et al., “Deep non-contact photoacoustic initial pressure imaging,” Optica 5(7), 814–820 (2018). 10.1364/OPTICA.5.000814

[15] Martell M. T. Haven N. J. Zemp R. J., “Multimodal imaging with spectral-domain optical coherence tomography and photoacoustic remote sensing microscopy,” Opt. Lett. 45(17), 4859–4862 (2020). OPLEDP 0146-9592 10.1364/OL.398940 32870876

[16] Zhou J. et al., “Dual-modal imaging with non-contact photoacoustic microscopy and fluorescence microscopy,” Opt. Lett. 46(5), 997–1000 (2021). OPLEDP 0146-9592 10.1364/OL.417273 33649646

[17] Kedarisetti P. et al., “Label-free lipid contrast imaging using non-contact near-infrared photoacoustic remote sensing microscopy,” Opt. Lett. 45(16), 4559–4562 (2020). OPLEDP 0146-9592 10.1364/OL.397614 32797009

[18] Wojtkowski M. et al., “Full range complex spectral optical coherence tomography technique in eye imaging,” Opt. Lett. 27(16), 1415–1417 (2002). OPLEDP 0146-9592 10.1364/OL.27.001415 18026464

[19] Nassif N. et al., “*In vivo* high-resolution video-rate spectral-domain optical coherence tomography of the human retina and optic nerve,” Opt. Express 12(3), 367–376 (2004). OPEXFF 1094-4087 10.1364/OPEX.12.000367 19474832

[20] Leitgeb R. Hitzenberger C. Fercher A. F., “Performance of Fourier domain vs. time domain optical coherence tomography,” Opt. Express 11(8), 889–894 (2003). OPEXFF 1094-4087 10.1364/OE.11.000889 19461802

[21] Yun S. et al., “High-speed spectral-domain optical coherence tomography at 1.3 *μ*m wavelength,” Opt. Express 11(26), 3598–3604 (2003). OPEXFF 1094-4087 10.1364/OE.11.003598 19471496 PMC2713046

[22] Laser Institute of America, “American national standard for safe use of lasers,” ANSI Z-136.1 (2007).

[23] Braaf B. et al., “Real-time eye motion correction in phase-resolved OCT angiography with tracking SLO,” Biomed. Opt. Express 4(1), 51–65 (2013). BOEICL 2156-7085 10.1364/BOE.4.000051 23304647 PMC3539196

